# Prevalence and characteristics of older adults with type 2 diabetes mellitus living in French Caribbean nursing homes: results from the baseline KASEHPAD study

**DOI:** 10.1007/s40520-025-03008-5

**Published:** 2025-03-24

**Authors:** Maturin Tabué-Teguo, Nadine Simo, Christine Rambhojan, Laurys Letchimy, Michel Bonnet, Fritz-Line Vélayoudom, Denis Boucaud-Maitre

**Affiliations:** 1 Centre Hospitalo-Universitaire de Martinique, Fort-de-France, Martinique France; 2 Equipe EPICLIV, Université Des Antilles, Fort-de-France, Martinique France; 3 Centre Hospitalo-Universitaire Guadeloupe, Pointe-à-Pitre, Guadeloupe France; 4 Department of Endocrinology-Diabetology, University Hospital of Guadeloupe, 97139 Les Abymes, France; 5 Inserm UMR1283, CNRS UMR8199, European Genomic Institute for Diabetes (EGID), Institut Pasteur de Lille, Lille, France; 6 Centre Hospitalier Le Vinatier, 95 Bd Pinel, 69678 Bron, France

**Keywords:** Diabetes, Nursing homes, Caribbean, Overtreatment

## Abstract

**Background:**

The management of older adults with type 2 diabetes mellitus (T2DM) has not been previously assessed in nursing homes within the Caribbean region.

**Aims:**

To investigate the prevalence of T2DM among residents of French Caribbean nursing homes and to characterize their health and functional status.

**Methods:**

This cross-sectional study is based on baseline screening data from the KASEHPAD (Karukera Study of Ageing in Nursing Homes) cohort. Clinical characteristics and standard geriatric parameters were systematically collected and analyzed. Hemoglobin A1c (HbA1c) levels of older adults with diabetes were retrospectively extracted from patient records.

**Results:**

A total of 332 participants aged 60 years or older were recruited from six nursing homes between September 2020 and November 2022. The prevalence of T2DM among residents was 28.3% (95% CI: 23.5–33.2). Among older adults with T2DM, 85.1% had hypertension, 17.1% had heart failure, and 24.1% had a history of stroke. The mean HbA1c level was 7.32 ± 1.5%. Of the 35 individuals (42.7%) with HbA1c < 7%, 19 (54.3%) were receiving antidiabetic medications. Multivariate analysis identified HbA1c as the sole factor significantly associated with antidiabetic medication use (OR: 1.76, 95% CI: 1.12–3.04).

**Discussion:**

Older adults with T2DM residing in French Caribbean nursing homes exhibit a high prevalence of cardiovascular risk factors and are at risk of overtreatment. The management of T2DM in this population appears to be driven primarily by blood glucose levels.

**Conclusion:**

As the prevalence of older adults with T2DM is expected to rise in the Caribbean region, this trend will present significant challenges in delivering tailored care within future nursing home settings.

## Introduction

The prevalence of type 2 diabetes mellitus (T2DM) exhibits significant variation across populations and clinical settings [1]. In France, the prevalence of T2DM is 4.6% in the general population, with rates increasing substantially with age. Approximately one in five men aged 70 to 85 years and one in seven women aged 75 to 85 years receive pharmacological treatment for T2DM [2]. The prevalence is notably higher in the French Caribbean islands, specifically Martinique and Guadeloupe, where it is approximately double that observed in mainland France [3]. According to estimates by the International Diabetes Federation, the age-adjusted prevalence of T2DM in the Caribbean is 9.6%; however, these estimates are derived from limited studies, as many Caribbean countries lack representative primary data (4). Reported prevalence rates include 4.9% in Barbados [4], 5.4% in Haiti [5], and up to 23% in St. Kitts and Nevis [6]. Across the region, the incidence of age-related adverse outcomes, such as chronic diseases (hypertension, diabetes, and cancer), dementia, and neuropsychiatric syndromes, is projected to rise significantly in the coming years [4]. The elevated burden of chronic diseases in the French Caribbean may be partially attributed to the unique history of its populations. Factors include genetic admixture, a transition toward sedentary lifestyles, cultural and dietary shifts, changing occupational environments, and epigenetic modifications. Black Caribbean populations are known to exhibit distinct diabetes profiles compared to other populations of African descent. For example, while T2DM spans the entire BMI range in African populations, it tends to be more severe in obese individuals of Caribbean descent. Additionally, individuals of Caribbean origin often experience poorer glycemic control, lower health literacy, and a higher incidence of complications compared to Africans or African Americans [7].

The medical and cultural contexts in the French Caribbean differ markedly from those in mainland France. In these territories, approximately 90% of the population is of African descent. Regarding T2DM and its associated risk factors, the average BMI is slightly lower than in mainland France (28.4 kg/m^2^ vs. 29.5 kg/m^2^), and tobacco and alcohol consumption are less prevalent. Glycemic control is generally poorer, with an average HbA1c of 7.5% compared to 7.2% in mainland France [3]. Severe complications further exacerbate the burden of diabetes in the French Caribbean. For instance, the rate of lower-limb amputations is 1.3 times higher, and hospitalizations for chronic kidney failure occur at twice the rate observed in mainland France, particularly in Martinique [8].

Older adults with T2DM are at greater risk of several clinical conditions, including malnutrition, infections, renal impairment, cognitive decline, depression, comorbidities, and polypharmacy [1, 9]. The management of T2DM in older adults has been the focus of several position statements issued by organizations such as the American Diabetes Association [1], the European Diabetes Working Party for Older People [10], and the International Diabetes Federation [11]. In France, the GERODIAB cohort study has provided valuable insights into diabetic complications, geriatric impairments, and mortality associated with glycemic control in older adults with T2DM. This longitudinal study followed nearly 1,000 elderly individuals with T2DM over a 5-year period [12]. Findings from the GERODIAB study indicated that an HbA1c level below 8.6% was associated with reduced mortality, and an acceptable target range of 5.8–6.7% was proposed, provided patients are not subjected to drug-induced hypoglycemia. However, the GERODIAB study did not include older adults residing in nursing homes or individuals living in the Caribbean, leaving significant gaps in knowledge about diabetes management in these specific populations. Standardized diabetes management for nursing home residents remains limited due to the heterogeneity of comorbidities and the lack of clinical trials tailored to this demographic [9].

To the best of our knowledge, no prior study has examined the prevalence of T2DM among older adults residing in nursing homes in the Caribbean. Notably, with the exception of the French West Indies, nursing homes are scarce in this region, and the care of older adults is predominantly provided by their families [13]. As the Caribbean grapples with the challenges of demographic transitions, including an increasing proportion of older adults [14], it is crucial to understand the healthcare trajectories of older adults within existing nursing homes and the broader context and challenges of dependency care across the Caribbean islands. This study aimed to estimate the prevalence of T2DM in nursing homes and to identify factors associated with diabetes treatment. To address this hypothesis, we utilized data from the KArukera Study of Aging in EHPAD (KASEHPAD), a longitudinal cohort study conducted in nursing homes in the French West Indies.

## Methods

### Study design

The KASEHPAD study was a prospective, observational study conducted across six nursing homes in Martinique and Guadeloupe (French West Indies). This study aimed to describe the health trajectories of older adults living in nursing homes over one year. Inclusion data have been previously published [14]. For this analysis, a cross-sectional approach was employed to evaluate the baseline characteristics of the study participants. The KASEHPAD study was approved by the EST 1 French Ethics Committee on June 2, 2020, and was registered on ClinicalTrials.gov on October 13, 2020 (NCT04587466).

### Data

At baseline, healthcare professionals conducted interviews with the participants and their professional caregivers. These interviews included comprehensive physical, cognitive, and psychological assessments of the participants. Professional caregivers provided detailed information on various aspects of the participants’ profiles, including sociodemographic characteristics, medical history, medication use, nutritional status, activity levels, and degree of dependence. Global cognitive function was assessed using the 30-item Mini-Mental State Examination (MMSE) [15]. Changes in physical function were evaluated using the basic Activities of Daily Living (ADL) score [16], while nutritional status was measured using the short form of the Mini Nutritional Assessment [17]. Quality of life was assessed through the Questionnaire on Quality of Life in Alzheimer’s Disease (QoL-AD) [18], and the risk of pressure ulcer development was evaluated using the Braden Scale [19]. Hemoglobin A1c (HbA1c) levels of older adults with diabetes were retrospectively extracted from patient records.

### Statistical analysis

Quantitative variables were expressed as means ± standard deviations, while qualitative variables were presented as percentages. Demographic and clinical characteristics were compared between older adults with and without type 2 diabetes mellitus (T2DM) using independent t-tests or Wilcoxon tests for continuous variables, and chi-squared or Fisher's exact tests for categorical variables. Similar analyses were conducted to compare older T2DM adults with and without diabetes medication. A logistic regression model was employed to identify predictors of diabetes medication use. A p-value of less than 0.05 was considered statistically significant. Missing data were not imputed. All statistical analyses were conducted using R software (version 3.0.2).

## Results

### Clinical characteristics

A total of 332 participants aged 60 years or older were recruited from six nursing homes between September 2020 and November 2022. The prevalence of type 2 diabetes mellitus (T2DM) was found to be 28.3% (95% CI: 23.5–33.2). The mean age of older adults with diabetes was 81.1 ± 10.0 years, and 57.5% of them were female.

When comparing older adults with or without T2DM (Table 1), older adults with T2DM had a significantly higher body mass index (BMI) (24.4 ± 5.3 vs. 22.4 ± 4.8, p < 0.001), as well as a higher prevalence of hypertension (85.1% vs. 59.2%, p < 0.001), heart failure (17.0% vs. 7.6%, p = 0.010), hypercholesterolemia (26.6% vs. 11.8%), stroke (24.5% vs. 13.1%, p = 0.011), kidney disease (22.3% vs. 11.3%, p = 0.010), and lower limb arteriopathy (16.0% vs. 8.4%, p = 0.043). In total, five older adults were on end-stage renal disease with dialysis: two were older adults with diabetes (2.1%) and three did not have diabetes (1.3%).

**Table 1:** Comparison of clinical characteristics between residents with or without T2DM

Characteristics	n	Mean ± CI or n (%)	Residents with T2DM (n = 94)	Other’s residents (n = 238)	p
Age	332	81.3 ± 10.1	81.1 ± 10.0	81.3 ± 10.1	0.877
Gender (men)	332	168 (50.6%)	40 (42.5%)	128 (53.8%)	0.065
BMI	287	23.0 ± 5.0	24.4 ± 5.3	22.4 ± 4.8	**0.003**
Length of stay in nursing homes (years)	307	4.4 ± 4.1	4.5 ± 4.7	4.4 ± 3.8	0.742
Hypertension (= yes)	332	221 (66.8%)	80 (85.1%)	141 (59.2%)	** < 0.001**
Hypercholesterolemia	332	53 (16.0%)	25 (26.6%)	28 (11.8%)	** < 0.001**
Cardiac failure	332	34 (10.2%)	16 (17.0%)	18 (7.6%)	**0.010**
Myocardial infarction	332	11 (3.3%)	5 (5.3%)	6 (2.5%)	0.304
Stroke	331	54 (16.3%)	23 (24.5%)	31 (13.1%)	**0.011**
Dementia	332	173 (52.1%)	50 (53.2%)	123 (51.7%)	0.804
Parkinson’s disease	332	30 (9.0%)	6 (6.4%)	24 (10.1%)	0.289
Depression	332	67 (20.2%)	15 (16.0%)	52 (21.8%)	0.228
Hemiplegia	331	32 (9.7%)	13 (13.8%)	19 (8.0%)	0.107
Kidney disease	332	48 (14.5%)	21 (22.3%)	27 (11.3%)	**0.010**
Neuropathy	332	19 (5.7%)	8 (8.5%)	11 (4.6%)	0.169
Low limb arteriopathy	332	35 (10.5%)	15 (16.0%)	20 (8.4%)	**0.043**
Cancer	330	14 (4.2%)	2 (2.1%)	12 (5.1%)	0.364

Bold values indicate p < 0.05

Regarding scores on geriatric assessment scales (Table 2), no significant differences were observed between groups in terms of cognition, dependence (including incontinence), quality of life, or the risk of developing a pressure ulcer. However, the malnutrition score was higher in T2DM patients (Mini Nutritional Assessment [MNA] score: 10.3 ± 3.5 vs. 9.4 ± 3.5, p = 0.046).

**Table 2:** Comparison of geriatric's scale scores between older adults with or without T2DM

Scale	All (n = 332)	Diabetic (n = 94)	Non Diabetic (n = 238)	p
MMSE (n = 295)	11.3 ± 9.4	10.4 ± 8.9	11.6 ± 9.6	0.318
MMSE ≤ 18	221 (74.9%)	67 (78.8%)	154 (73.3%)	0.324
MNA (n = 319)	9.7 ± 3.5	10.3 ± 3.5	9.4 ± 3.5	0.046
MNA score ≤ 7	88 (27.6%)	19 (21.1%)	69 (30.1%)	0.104
ADL (n = 326)	2.4 ± 2.1	2.1 ± 2.0	2.5 ± 2.1	0.146
*full assistance for bathing*	209 (64.1%)	61 (66.3%)	148 (63.2%)	0.605
*Full assistance of dressing*	208 (63.8%)	61 (66.3%)	147 (62.8%)	0.556
*full assistance for toileting*	157 (48.2%)	48 (52.2%)	109 (46.6%)	0.363
*Bedridden*	74 (22.7%)	25 (27.2%)	49 (20.9%)	0.226
*Incontinence*	176 (54.0%)	54 (58.7%)	122 (52.1%)	0.285
*Totally dependent at meals*	110 (33.7%)	36 (39.1%)	74 (31.6%)	0.197
QOL-AD (n = 110)	29.2 ± 8.2	27.6 ± 8.8	30.0 ± 7.4	0.129
Braden (n = 321)	18.4 ± 3.8	18.0 ± 4.2	18.5 ± 3.7	0.344

### Glycemic control and diabetes medication

The HbA1c measurement was available for 82 residents with type 2 diabetes mellitus (T2DM). The mean HbA1c was 7.32% ± 1.5%, with 35 participants (42.7%) exhibiting an HbA1c level of < 7% (Table 3). Among the 35 participants with HbA1c < 7%, 19 (54.3%) were being treated, mainly by insulin (n = 12, 63.2%) or liraglutide (n = 3, 15,8%).

**Table 3:** Glycemic control and drug treatment of older adults with T2DM living in French Caribbean nursing homes

HbA1c (n = 82)	
Mean	7.32 ± 1.5
Median	7.15
< 7%	35 (42.7%)
≥ 7 and < 8.5%	29 (35.4%)
≥ 8.5%	18 (21.9%)
Diabetes treatment (n = 94)	
No treatment, n (%)	35 (37.2%)
OAD, n (%)	24 (25.5%)
*Metformin*	19 (20.2%)
*Glinides*	1 (1.1%)
*Sulfonylureas*	2 (2.1%)
*DPP4 inhibitors*	6 (6.4%)
GLP1 agonist	7 (7.4%)
Insulin, n (%)	41 (43.6%)
Drug combination, n (%)	15 (16.0%)
OAD only	14 (14.9%)
Insulin only	31 (33.0%)
OAD-Insulin combination	7 (7.4%)
GLP-1 – Insulin combination	3 (3.2%)
OAD-GLP-1	5 (5.3%)

Among the 94 residents with diabetes, 37.2% were not receiving any antidiabetic treatment, 43% were on insulin, and 25% were receiving oral antidiabetic agents. Metformin was prescribed to 20.2% of older adults with T2DM, while only two individuals were prescribed sulfonylureas.

When analyzing the factors associated with antidiabetic treatment (Table 4), bivariate analysis revealed that individuals receiving antidiabetic treatment were younger, more often male, had a higher BMI, and exhibited a higher prevalence of hypertension and hypercholesterolemia. Additionally, they demonstrated better physical performance (lower dependence), improved nutritional status, and a higher HbA1c (7.7% vs. 6.7%; p = 0.002).

**Table 4:** Comparison of older adults taking or not taking antidiabetic drugs

			Bivariate analysis	Multivariate analysis	
Characteristics	Diabetes medication (n = 59)	No diabetes medication (n = 35)	p	p	OR (CI95%)
Age (n = 94)	78.8 ± 9.0	85.0 ± 10.4	0.003	0.338	0.96 (0.88–1.04)
Gender (men) (n = 94)	30 (50.8%)	10 (28.6%)	0.034	0.568	1.51 (0.36–6.47)
BMI (n = 79)	25.7 ± 5.5	21.7 ± 3.7	0.001	0.165	1.12 (0.96–1.33)
Hypertension	53 (89.8%)	27 (77.1%)	< 0.001	0.864	0.85 (0.14–4.96)
Cardiac failure	9 (15.2%)	7 (20.0%)	0.554	–	–
Stroke	17 (28.8%)	6 (17.1%)	0.203	–	–
Hypercholesterolemia	20 (33.9%)	7 (20.0%)	0.037	0.319	2.06 (0.52–9.39)
Kidney disease	15 (25.4%)	6 (17.1%)	0.351	–	–
Low limb arteriopathy	10 (16.9%)	5 (14.3%)	0.733	–	–
Dementia	28 (47.5%)	22 (62.9%)	0.148	–	–
MNA score (n = 90)	11.1 ± 3.3	8.9 ± 3.4	0.004	0.916	1.01 (0.81–1.27)
ADL score (n = 92)	2.5 ± 2.0	1.4 ± 1.8	0.009	0.516	1.12 (0.79–1.56)
HbA1C score (n = 82)	7.7 ± 1.6	6.7 ± 1.3	0.002	0.022	1.76 (1.12–3.04)

In multivariate analysis (n = 71), adjusting for age, sex, BMI, hypertension, hypercholesterolemia, dementia, MNA score, ADL score, and HbA1c, only HbA1c was significantly associated with antidiabetic treatment (OR: 1.76; 95% CI: 1.12–3.04).

## Discussion

In our study, we observed a T2DM prevalence of 28.3%. This prevalence is notably high compared to the 10–20% reported in other French nursing homes [20, 21], but is consistent with the figures reported in U.S. nursing homes [22]. This higher prevalence can likely be attributed to the elevated rates of T2DM in Caribbean territories, which are twice as high as in mainland France. Residents with T2DM in our study were of an age comparable to other residents, whereas international data generally suggest that individuals with diabetes in nursing homes tend to be younger [23]. A significant proportion of T2DM residents were women (57.5%), which reflects a unique demographic characteristic of the French West Indies compared to global data [24]. Caribbean individuals with diabetes also exhibited a markedly high co-occurrence of hypertension, with 85% of T2DM residents being hypertensive—double the rates reported in nursing homes across Europe [23]. The prevalence of macrovascular complications was also high, with a quarter of residents with T2DM reporting a history of stroke. Although the prevalence of dependence, cognitive impairment, and nutritional alterations did not differ significantly between T2DMand non-T2DM residents, the rate of malnutrition among older adults with T2DM was notably high (21.1%). Malnutrition has been shown to significantly affect quality of life, particularly in nursing home populations [25]. Lastly, 78.8% of T2DM residents exhibited major cognitive impairments (MMSE ≤ 18), and 53.3% had a diagnosis of dementia, indicating that cognitive function may be under-evaluated in nursing home settings.

Regarding treatment, 37.2% of residents with T2DM were not receiving any antidiabetic medications. Factors associated with the absence of treatment included advanced age and higher levels of dependence. In this population, with increasing age and comorbidities, strict control of type 2 diabetes mellitus (T2DM) takes a secondary role to the prevention of fall risks, malnutrition, sarcopenia, cognitive decline, and acute metabolic events. Nevertheless, 42% of older adults with T2DM had an HbA1c < 7%, with more than half receiving treatment, mainly insulin. These findings are consistent with other studies [26–28], which suggest potential overtreatment of T2DM individuals in nursing homes. In this frail and functionally dependent population, clinical guidelines for older adults with T2DM in nursing homes emphasize the avoidance of hypoglycemia [9]. However, over 40% of treatments involved insulin or sulfonylureas. Furthermore, multivariate analysis indicated that the use of antidiabetic medications was primarily determined by HbA1c levels, whereas European [10] and American (10) guidelines recommend that glycemic targets should consider coexisting chronic conditions, cognitive impairments, and functional limitations. Dipeptidyl peptidase-4 (DPP-4) inhibitors and glucagon-like peptide-1 (GLP-1) analogs were rarely prescribed. Additionally, sodium-glucose cotransporter-2 inhibitors (SGLT-2is) were introduced to the French market relatively late (March 2021) [29], which likely explains the absence of their use in this population.

The strength of this study lies in its first-time description of the clinical characteristics of nursing home residents in the Caribbean. Outside of the French West Indies, only Jamaica has implemented nursing homes within the region. The French Caribbean serves as a bridge between other Caribbean countries in terms of ethnology and socio-cultural aspects, as well as with high-income nations due to its affiliation with France. Consequently, it represents an interesting territory for research and experimentation, providing an opportunity to test solutions in a region experiencing an acceleration in population aging. The data from this study suggest that older adults with T2DM may not be adequately monitored by specialists, as evidenced by the observed HbA1c levels and the therapies administered. However, our study has several limitations. It is an epidemiological study, and its primary objective was not specifically focused on T2DM. As a result, a detailed analysis of microvascular complications, such as renal function stages or retinopathy, was not conducted. Additionally, fasting blood glucose levels, the use of capillary point-of-care monitoring, and occurrences of hypoglycemia were not recorded.

## Conclusion

T2DM is particularly prevalent in French Caribbean nursing homes. Glycemic control among residents appeared excessively stringent for the majority, with the prescription of antidiabetic medications primarily driven by blood glucose levels. Furthermore, insulin was widely prescribed, heightening the risk of hypoglycemia. As the prevalence of older adults with T2DM is anticipated to increase in the Caribbean, this will present significant challenges in adapting nursing home care for this population, particularly given the scarcity of specialists trained in diabetes management. Consequently, it is crucial to understand the unique characteristics of these territories to implement appropriate interventions and enhance clinical practices.

## Data Availability

No datasets were generated or analysed during the current study.

## References

[1] American Diabetes Association Professional Practice Committee (2024) 13. Older adults: standards of care in diabetes-2024. Diabetes Care 47:S244–S257. 10.2337/dc24-S013 38078580 10.2337/dc24-S013 PMC10725804

[2] Fuentes S, Mandereau-Bruno L, Regnault N et al (2020) Is the type 2 diabetes epidemic plateauing in France? A nationwide population-based study. Diabetes Metab. 10.1016/j.diabet.2019.12.006 31923577 10.1016/j.diabet.2019.12.006

[3] Hernandez H, Piffaretti C, Gautier A, et al (2023) Prévalence du diabète connu dans 4 départements et régions d’outre-mer : Guadeloupe, Martinique, Guyane et La Réunion. Résultats du Baromètre de Santé publique France de 2021. Bull Épidémiol Hebd (20–21):424–31. http://beh.santepubliquefrance.fr/beh/2023/20-21/2023_20-21_2.html

[4] Yisahak SF, Beagley J, Hambleton IR et al (2014) IDF Diabetes Atlas. Diabetes in North America and the Caribbean: an update. Diabetes Res Clin Pract 103:223–230. 10.1016/j.diabres.2013.11.009 24321468 10.1016/j.diabres.2013.11.009

[5] Sufra R, Lookens Pierre J, Dade E et al (2022) Diabetes epidemiology among adults in Port-au-Prince, Haiti: a cross-sectional study. Front Endocrinol (Lausanne). 13:841675. 10.3389/fendo.2022.841675 35282460 10.3389/fendo.2022.841675 PMC8913034

[6] Richardson JW, Kelly KD, Kumodzi TK et al (2019) Type 2 diabetes prevalence, distribution and risk factors in St. Kitts and Nevis, West Indies. Int J Diabetes Clin Res 6:114. 10.23937/2377-3634/1410114 31934633 10.23937/2377-3634/1410114 PMC6957082

[7] Utumatwishima JN, Chung ST, Bentley AR et al (2018) Reversing the tide—diagnosis and prevention of T2DM in populations of African descent. Nat Rev Endocrinol 14:45–56. 10.1038/nrendo.2017.127 29052590 10.1038/nrendo.2017.127

[8] Fosse-Edorh S, Lavalette C, Piffaretti C et al (2023) Caractéristiques, état de santé et recours aux soins des personnes présentant un diabète de type 2 résidant en outre-mer : résultats de l’étude Entred 3. Bull Épidémiol Hebd (20–21):412–23. https://beh.santepubliquefrance.fr/beh/2023/20-21/2023_20-21_1.html

[9] Idrees T, Castro-Revoredo IA, Migdal AL et al (2022) Update on the management of diabetes in long-term care facilities. BMJ Open Diab Res Care 10:e002705. 10.1136/bmjdrc-2021-002705 35858714 10.1136/bmjdrc-2021-002705 PMC9305812

[10] Sinclair AJ, Paolisso G, Castro M et al (2011) European diabetes Working Party for older people 2011 clinical guidelines for type 2 diabetes mellitus. executive summary. Diabetes Metab 37:S27-38 22183418 10.1016/S1262-3636(11)70962-4

[11] International Diabetes Federation. Recommendations for managing type 2 diabetes in primary care, 2017. Available: www.idf.org/managing-type2-diabetes2021

[12] Doucet J, Verny C, Balkau B et al (2018) Haemoglobin A1c and 5-year all-cause mortality in French type 2 diabetic patients aged 70 years and older: The GERODIAB observational cohort. Diabetes Metab 44:465–472. 10.1016/j.diabet.2018.05.003 29859993 10.1016/j.diabet.2018.05.003

[13] Boucaud-Maitre D, Simo N, Villeneuve R et al (2024) Clinical profiles of older adults in French Caribbean nursing homes: a descriptive cross-sectional study. Front Med (Lausanne) 11:1428443. 10.3389/fmed.2024.1428443 39355845 10.3389/fmed.2024.1428443 PMC11442247

[14] Ibáñez A, Pina-Escudero SD, Possin KL et al (2021) Multi-partner consortium to expand dementia research in Latin America. Dementia caregiving across Latin America and the Caribbean and brain health diplomacy. Lancet Healthy Longev 2:e222–e231. 10.1016/S2666-7568(21)00031-3 34790905 10.1016/s2666-7568(21)00031-3 PMC8594860

[15] Folstein MF, Folstein SE, McHugh PR (1975) “Mini-mental state”. A practical method for grading the cognitive state of patients for the clinician. J Psychiatr Res 12:189–198. 10.1016/0022-3956(75)90026-6 1202204 10.1016/0022-3956(75)90026-6

[16] Katz S, Ford AB, Moskowitz RW et al (1963) Studies of illness in the aged. The index of adl: a standardized measure of biological and psychosocial function. JAMA 185:914–919. 10.1001/jama.1963.03060120024016 14044222 10.1001/jama.1963.03060120024016

[17] Vellas B, Guigoz Y, Garry PJ (1999) The mini nutritional assessment (MNA) and its use in grading the nutritional state of elderly patients. Nutrition 15:116–122. 10.1016/s0899-9007(98)00171-3 9990575 10.1016/s0899-9007(98)00171-3

[18] Logsdon RG, Gibbons LE, McCurry SM et al (1999) Quality of life in Alzheimer’s disease: patient and caregiver reports. J Ment Health Aging 5:21–32

[19] Prevention and treatment of pressure ulcers: quick reference guide. National Pressure Ulcer Advisory Panel, European Pressure Ulcer Advisory Panel and Pan Pacific Pressure Injury Alliance. 2014. URL: https://www.epuap.org/wp-content/uploads/2016/10/quick-reference-guide-digital-npuap-epuap-pppia-jan2016.pdf [accessed 2024–01–09]

[20] Rolland Y, van Abellan Kan G, Hermabessiere S et al (2009) Descriptive study of nursing home residents from the REHPA network. J Nutr Health Aging 13:679–683. 10.1007/s12603-009-0197-419657550 10.1007/s12603-009-0197-4

[21] Gayot C, Laubarie-Mouret C, Zarca K et al (2022) Effectiveness and cost-effectiveness of a telemedicine programme for preventing unplanned hospitalisations of older adults living in nursing homes: the GERONTACCESS cluster randomized clinical trial. BMC Geriatr 22:991. 10.1186/s12877-022-03575-6 36550496 10.1186/s12877-022-03575-6 PMC9773573

[22] Munshi MN, Florez H, Huang ES et al (2016) Management of diabetes in long-term care and skilled nursing facilities: a position statement of the American diabetes association. Diabetes Care 39:308–318 26798150 10.2337/dc15-2512 PMC5317234

[23] Szczerbińska K, Topinková E, Brzyski P et al (2015) The characteristics of diabetic residents in European nursing homes: results from the SHELTER study. J Am Med Dir Assoc 16:334–340. 10.1016/j.jamda.2014.11.009 25533147 10.1016/j.jamda.2014.11.009

[24] Kautzky-Willer A, Leutner M, Harreiter J (2023) Sex differences in type 2 diabetes. Diabetologia 66:986–1002. 10.1007/s00125-023-05891-x. (**published correction appears in Diabetologia. 2023 Jun;66(6):1165**) 37042956 10.1007/s00125-023-05913-8 PMC10163122

[25] Dorner B, Friedrich EK, Posthauer ME (2010) Practice paper of the American Dietetic Association: individualized nutrition approaches for older adults in health care communities. J Am Diet Assoc 110:1554–1563. 10.1016/j.jada.2010.08.023 20882715 10.1016/j.jada.2010.08.023

[26] Quilot E, Petit JM, Vergès B et al (2020) Are older patients with diabetes still being overtreated in French long-term care homes? Age Ageing 49:878–882. 10.1093/ageing/afaa051 32457990 10.1093/ageing/afaa051

[27] Lipska KJ, Ross JS, Miao Y et al (2015) Potential overtreatment of diabetes mellitus in older adults with tight glycemic control. JAMA Intern Med 175:356–362 25581565 10.1001/jamainternmed.2014.7345 PMC4426991

[28] Stasinopoulos J, Wood SJ, Bell JS et al (2021) Potential overtreatment and undertreatment of type 2 diabetes mellitus in long-term care facilities: a systematic review. J Am Med Dir Assoc 22:1889-1897.e5. 10.1016/j.jamda.2021.04.013 34004183 10.1016/j.jamda.2021.04.013

[29] De Germay S, Pambrun E, Pariente A et al (2024) Use of sodium-glucose cotransporter-2 inhibitors in France: analysis of french nationwide health insurance database. Diabetes Obes Metab. 10.1111/dom.15472 38288619 10.1111/dom.15472

